# Transcranial direct current stimulation (tDCS) reduces motivation to drink ethanol and reacquisition of ethanol self-administration in female mice

**DOI:** 10.1038/s41598-021-03940-2

**Published:** 2022-01-07

**Authors:** Solène Pedron, Stéphanie Dumontoy, Maria del Carmen González-Marín, Fabien Coune, Andries Van Schuerbeek, Emmanuel Haffen, Mickael Naassila, Vincent Van Waes

**Affiliations:** 1 Laboratoire de Recherches Intégratives en Neurosciences et Psychologie Cognitive UR-LINC 481, Université Bourgogne Franche-Comté, Besançon, France; 2 INSERM UMR 1247 – Research Group on Alcohol and Pharmacodependences (GRAP), Université de Picardie Jules Verne, Amiens, France; 3 Department of Pharmaceutical Sciences, Research Group Experimental Pharmacology, Center for Neurosciences (C4N), Vrije Universiteit Brussel, Brussels, Belgium

**Keywords:** Neuroscience, Motivation, Reward, Preclinical research

## Abstract

Transcranial direct current stimulation (tDCS) is an emerging noninvasive brain neuromodulation technique aimed at relieving symptoms associated with psychiatric disorders, including addiction. The goal of the present study was to better identify which phase of alcohol-related behavior (hedonic effect, behavioral sensitization, self-administration, or motivation to obtain the drug) might be modulated by repeated anodal tDCS over the frontal cortex (0.2 mA, 20 min, twice a day for 5 consecutive days), using female mice as a model. Our data showed that tDCS did not modulate the hedonic effects of ethanol as assessed by a conditioned place preference test (CPP) or the expression of ethanol-induced behavioral sensitization. Interestingly, tDCS robustly reduced reacquisition of ethanol consumption (50% decrease) following extinction of self-administration in an operant paradigm. Furthermore, tDCS significantly decreased motivation to drink ethanol on a progressive ratio schedule (30% decrease). Taken together, our results show a dissociation between the effects of tDCS on “liking” (hedonic aspect; no effect in the CPP) and “wanting” (motivation; decreased consumption on a progressive ratio schedule). Our tDCS procedure in rodents will allow us to better understand its mechanisms of action in order to accelerate its use as a complementary and innovative tool to help alcohol-dependent patients maintain abstinence or reduce ethanol intake.

## Introduction

Addiction is a chronically relapsing disorder characterized by (i) a compulsion to seek and take a particular drug despite knowledge of adverse consequences, (ii) loss of the ability to self-regulate intake, and (iii) the appearance of a negative emotional state when access is prevented^1–5^. Alcohol use disorder (AUD) affects more than 2 billion people and accounts for nearly 6% of all deaths worldwide^6^. Excessive levels of ethanol consumption and a high rate of relapse represent specific characteristics of AUD^7,8^. To date, there are five medications with marketing authorization in France for treating alcohol dependence: acamprosate, disulfiram, baclofen, nalmefene and naltrexone. These medications, when used in combination with psychological intervention, can help alcohol-dependent subjects maintain abstinence or reduce their desire to drink^9^. However, they have low to moderate effect sizes, and although effective for some individuals, there is currently no single treatment that works for all patients. Moreover, these molecules can also have deleterious effects on sleep^10^.

Ethanol, like other drugs of abuse, involves mediation of the brain reward circuitry, particularly the prefrontal cortex (PFC), which plays an important role in higher-order executive functions (*e.g.,* salience attribution). The PFC also participates in the maintenance of drug addiction by driving relapse to drug taking elicited by drug-associated cues^11,12^. Neuromodulation of the PFC might therefore be of interest in treating AUD. Among the noninvasive modulatory techniques currently available, transcranial direct current stimulation (tDCS) uses a weak constant electrical current to modulate the excitability and activity of specific areas of the cerebral cortex. The advantages of tDCS are compelling; most notably, it is noninvasive, easy to use, low cost, and has limited side effects^13–15^. tDCS is well tolerated both in clinical populations and in healthy subjects^16–20^. An increasing number of clinical studies indicate that the application of tDCS over the dorsolateral prefrontal cortex (DLPFC) decreases drug craving in chronic users^14^. For example, tDCS attenuates both smoking behavior and attention to smoking-related cues^21–27^ and reduces the craving for several drugs of abuse such as marijuana^28^, methamphetamine^29–31^, and alcohol in heavy drinkers and alcohol-dependent subjects^19,20,32–42^. The mechanisms underlying these beneficial effects, however, remain poorly known.

We have recently developed a procedure to apply tDCS in rodents to study its behavioral and neurobiological effects^43–47^; see^48^ for a detailed procedure. Our early studies showed that in mice, repeated anodal tDCS over the frontal cortex reduced nicotine-induced place preference conditioning as well as abnormal behaviors associated with chronic exposure to nicotine during adolescence^43^. We also found that tDCS produced long-lasting attenuation of cocaine-induced behavioral responses and gene regulation in corticostriatal circuits^44^. The goal of the present study was to extend these data and evaluate the efficacy of repeated tDCS treatment in animal models of ethanol exposure that reflect different aspects of alcohol consumption. In the first experiment, we used the place preference conditioning paradigm to evaluate the impact of tDCS on the hedonic effect of ethanol (“Do I like it?”). In a second experiment, we evaluated the effects of tDCS on the expression of ethanol-induced behavioral sensitization, which has been hypothesized to reflect drug-induced long-term neuroplasticity in the nucleus accumbens^49^. In these experiments, ethanol was passively administered, *i.e.,* intraperitoneally administered by the experimenter. Finally, we tested whether repeated tDCS treatment might facilitate the extinction and/or decrease the reacquisition of ethanol consumption in an operant self-administration paradigm. The motivational component (“How hard I am willing to work to obtain a dose of ethanol?”) was finally evaluated using a progressive ratio schedule. In this experiment, the consumption of ethanol was voluntary, *i.e.,* the mice controlled their oral consumption.

## Materials and methods

### Animals

Female mice were housed at six to eight per cage (except during surgery, recovery, and electrical stimulation periods, during which they were individually housed) under a 12-h light/dark cycle (lights on from 07:00 to 19:00 h) at a controlled temperature (21 ± 0.5 °C) and humidity (55 ± 10%). Experiments were conducted during the light phase of the cycle. Food and water were available ad libitum (unless otherwise indicated). Female rather than male mice were used in the present work because we used females in our previous tDCS studies^43,44^ and because we have already collected a significant quantity of data on alcohol-induced behavioral sensitization in female mice^50–53^. Moreover, female mice were used to ensure consistency with the majority of ethanol sensitization studies and because female rodents generally show increased susceptibility to drug-induced sensitization compared to males^54^. To the best of our knowledge, there are no studies to date showing a differential effect of tDCS between male and female mice. However, several groups have reported differential impact of tDCS in men and women (with the effects often being more marked in women than in men), which they have linked to factors such as sex hormones, anatomical differences, and neuroplasticity^55–62^.

All experimental procedures were performed in strict accordance with the Guide for the Care and Use of Laboratory Animals (NIH), the Animal Research: Reporting of In Vivo Experiments (ARRIVE) guidelines and the European Union regulations on animal research (Directive 2010/63/EU). They were approved by the University of Franche-Comté Animal Care and Use Committee (CEBEA-58) and the local ethics committees of Amiens (CREMEAP-C2EA96).

### Transcranial direct current stimulation

#### Surgery

**Figure 1:**
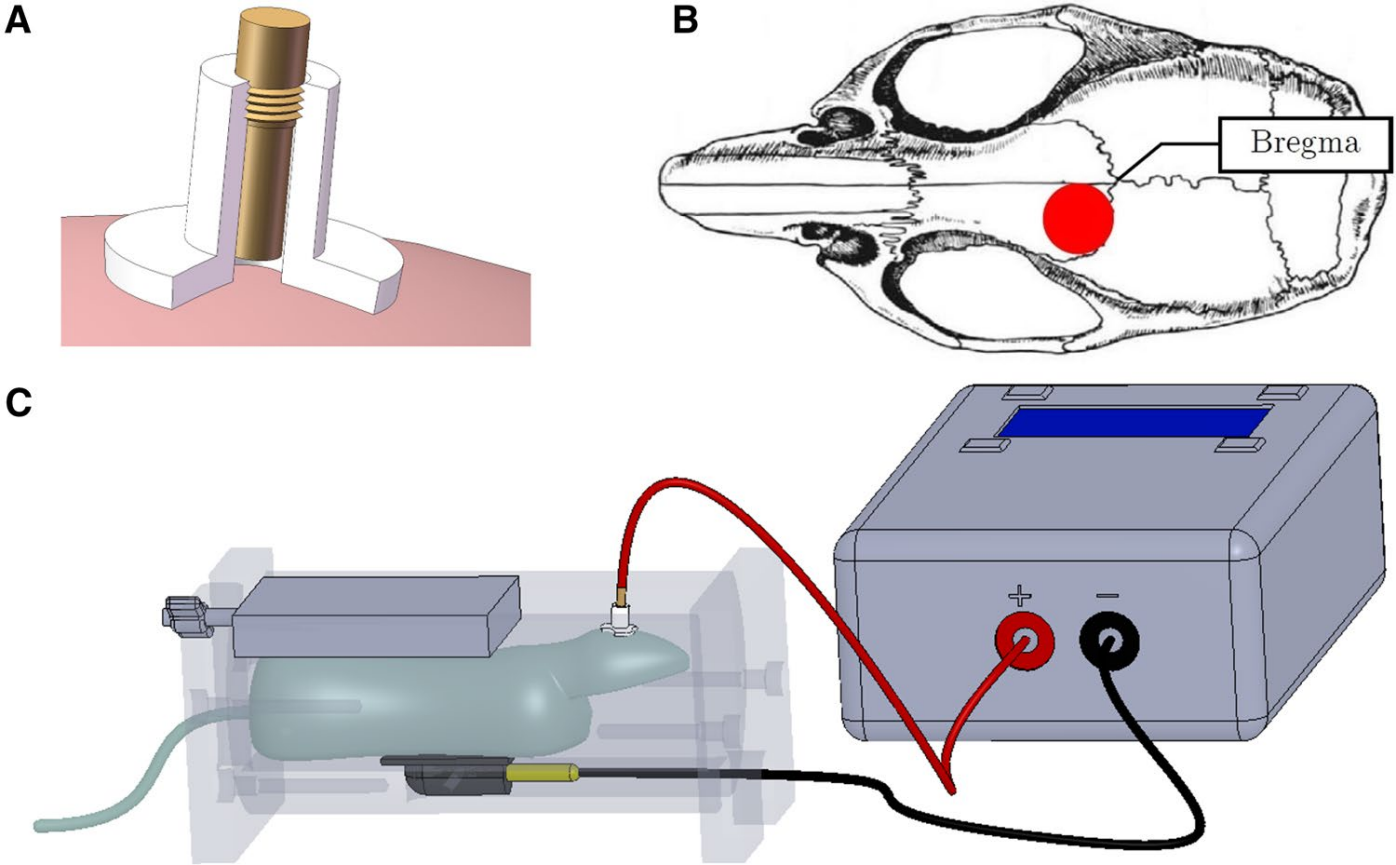
Illustration of the tDCS device used to deliver the current stimulation. (**A**) The electrode holder (internal diameter: 2.1 mm) was surgically affixed to the skull and filled with saline solution before stimulation. The stimulation electrode was screwed into the tubular plastic jacket so that it dipped into the saline solution. Only the saline solution was in contact with the skull. (**B**) The center of the electrode holder was positioned 1 mm anterior and 1 mm left of bregma (image adapted from Paxinos and Franklin^84^). (**C**) The mouse was placed in a custom-made restraint box. The anode (contact area 3.5 mm^2^) was positioned over the left frontal cortex, and the cathode (rubber-plate electrode, 9.5 cm^2^) was placed on the ventral thorax. A constant current of 0.2 mA was applied transcranially in 2 sessions × 20 min per day for five consecutive days, with a linear fade-in/fade-out of 10 s, using a direct current stimulator (DC-Stimulator Plus) or an Open-tES stimulator specifically designed for rodent research^45^.

Prior to surgery, mice were allowed 1 week of acclimation to the animal facility, during which time, they were repeatedly handled. A tubular plastic electrode holder (internal diameter 2.1 mm; DIXI Medical, France; Fig. 1A) was surgically affixed to the skull of each mouse. The animals were anesthetized with ketamine hydrochloride/xylazine (80/12 mg/kg; intraperitoneal (i.p.) injection) and were placed in a stereotaxic apparatus. The center of the electrode holder was positioned over the left frontal cortex, 1 mm rostral and 1 mm left of bregma, and fixed with glass ionomer cement (GC Fuji I, Leuven, Belgium; Fig. 1B)^43,44,48^. The animals were allowed 1 week to recover from surgery.

#### Stimulation protocol

The electrode holder was filled with saline (NaCl 0.9%), and the stimulation electrode (anode, diameter: 2.1 mm; DIXI Medical) was screwed into the electrode holder. A larger rectangular rubber-plate electrode (cathode, 9.5 cm^2^; Physiomed Elektromedizin AG, Schnaittach, Germany) was used as a counter electrode and was placed onto the ventral thorax (Fig. 1C)^43,44,48^. For 5 consecutive days, an anodal constant current (0.2 mA; 2 × 20 min/day, 5-h interstimulation interval, linear fade-in/fade-out: 10-s ramp) was transcranially applied over the frontal cortex using a DC-Stimulator Plus (NeuroConn, Ilmenau, Germany) or an Open-tES stimulator specifically designed for rodent research^45^. The animals were restrained and awake during tDCS to prevent a possible interaction between tDCS and anesthetic drugs. Control (sham) animals were subjected to the same procedure (surgery, restraining box, and electrode fixation), but no current was delivered. An important consideration is the extent to which our animal stimulation paradigm is equivalent to protocols used in humans. Indeed, our stimulation protocol is the same as that used in clinical trials in terms of time, length, and number of repetitions, but our protocol has a lower intensity: 0.2 mA *vs.* 2 mA. However, the current density was much higher in our animal model than in human protocols (57.1 in our model *vs.* 0.57 A/m^2^ for clinical trials) due to the smaller size of the electrode used for mice. This is of importance because the area stimulated by the current might be significantly different in mice and in humans, especially if considered relative to the size of the brain.

Liebetanz and collaborators found that lesions began to be induced after tDCS at a current density of 142.9 A/m^2^^63^. Jackson and collaborators^64^ found that when rats were stimulated using a 5.3 mm^2^ electrode (anode), lesions began to appear at 0.5 mA (current density: 94.2 A/m^2^). Another team^65^ published evidence that higher intensities (up to 1 mA, corresponding to a current density of 80 A/m^2^) are effective and safe. Even if there are some differences between our stimulation protocol and those cited above (*e.g.* polarity, rats *vs*. mice), we estimate that our stimulation protocol (current density: 57.1 A/m^2^) is safe.

In the present study, only one current intensity and one polarity were tested (0.2 mA, anodal stimulations). In a previous study by our group, we tested distinct current intensities and polarities^46^; our results indicated that the behavioral effect of tDCS (on depression-related behavior) was absent at intensities of 0.025 and 0.1 mA and emerged when the intensity was increased to 0.2 mA. At a current intensity of 0.2 mA, only anodal stimulation affected depression-like behavior; cathodal stimulation had no effect.

## Experiment 1: tDCS effects on ethanol-induced conditioned place preference

### Animals

Fifty-four female Swiss mice (8 weeks at the beginning of the experiment; Janvier Labs, France) were divided into six experimental groups: Sham-vehicle (veh) (N = 10), tDCS-veh (N = 10), Sham-1 g/kg (N = 10), tDCS-1 g/kg (N = 10), Sham-2 g/kg (N = 7), and tDCS-2 g/kg (N = 7). Swiss mice were chosen because of their interindividual variability, which models a heterogeneous population, and because we had previously used this strain in our experiments exploring the effects of tDCS^43,44^.

### Place preference test

**Figure 2:**
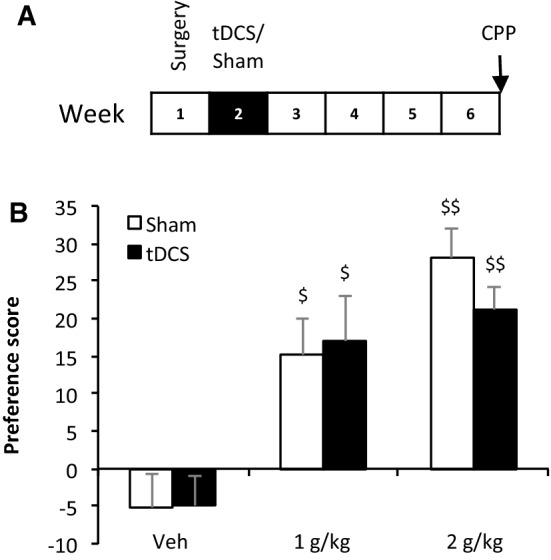
tDCS does not modulate ethanol-induced CPP. (**A**) Experimental design. (**B**) tDCS has no impact on ethanol-induced CPP (stimulation effect: F_(1, 48)_ = 33.7, *P* = 0.68). Ethanol induces a place preference similar in the sham and tDCS groups, irrespective of the ethanol dose tested (^$^*P* ≤ 0.05 and ^$$^*P* ≤ 0.01 *vs.* 0).

The animals were subjected to the conditioned place preference test (CPP) 4 weeks after the last electrical stimulation, as previously described^43,44^ (Fig. 2A). In this test, ethanol is passively administered, *i.e.,* intraperitoneally administered by the experimenter. Two doses of ethanol were tested (1 and 2 g/kg, i.p.). Ethanol solution was diluted in sterile physiological saline (0.9%) from 96% ethanol to reach a concentration of 20% (VWR-Prolabo, Fontenay-sous-Bois, France). Two groups received vehicle injections (*i.e.,* physiological saline) in both compartments and were used as control groups (Sham-veh and tDCS-veh).

Briefly, the CPP apparatus consisted of two main compartments (18 cm tall × 18 cm long × 24 cm deep, manufactured in house) linked by a corridor and displayed different features, both visual (walls: plain or hatched pattern) and tactile (floor texture: smooth or textured, respectively). On day 1 (preconditioning, D1), mice were placed in the corridor and allowed free access to the compartments for 10 min. The time spent in each compartment was recorded using the EthoVision system (Noldus, the Netherlands). On days 2–4 (conditioning phase, D2-D4), the mice received an injection of ethanol and an injection of vehicle daily (interval between injections: 6 h). After the injection, the mice were immediately confined in one of the two conditioning compartments for 15 min (the drug was always paired with the less preferred of the two compartments as measured at D1). On day 5 (postconditioning, D5), the mice were again allowed free access to both compartments for 10 min without any drug injection. The percentage of the time spent in the drug-paired compartment was calculated for the preconditioning (%D1) and postconditioning (%D5) phases as follows: drug-paired compartment (seconds)/(drug-paired compartment + vehicle-paired compartment (seconds)) × 100. Preference scores were then calculated as follows: %D5–%D1. The drug induced a conditioned place preference (*i.e*., a rewarding pleasant effect) if the preference score was significantly superior to 0 and a conditioned place aversion (an aversive unpleasant effect) if the preference score was significantly inferior to 0. A preference score not significantly different from 0 indicated that the injected substance had neither rewarding nor aversive effects.

## Experiment 2: tDCS effects on ethanol-induced behavioral sensitization

### Animals

Forty-seven female DBA/2 J mice were used (8 weeks old at the beginning of the experiment; Janvier, France). This strain was chosen for its high sensitivity to the stimulatory and sensitizing effects of ethanol on locomotion^66,67^.

### Locomotor activity cages

Locomotor activity was assessed using an infrared actimeter (LE 8811 Model, 45-cm width × 45-cm depth × 20-cm height, Bioseb, Chaville/Vitrolles, France). Each frame of the six locomotor activity cages was equipped with 16 × 16 infrared photocell beams (2 cm above the floor) and located in a dark experimental room (indirect 20-lx white light) isolated from external noise. Horizontal locomotion was measured by determination of photobeam breaks using ActiTrack software (Bioseb, France).

### Ethanol sensitization

**Figure 3:**
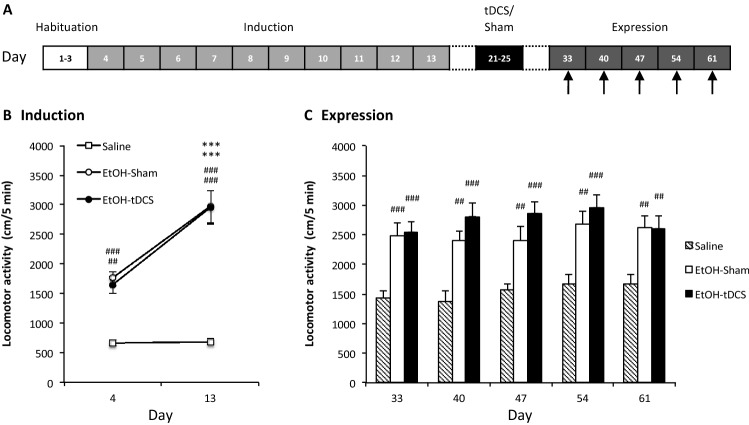
tDCS has no impact on the expression of ethanol-induced behavioral sensitization. (**A**) Experimental design. (**B**) Induction of behavioral sensitization to ethanol (before tDCS) (^##^*P* ≤ 0.01 and ^###^*P* ≤ 0.001, ethanol *vs.* saline; ***P ≤ 0.001, day 13 *vs.* day 4). (**C**) tDCS does not modulate the expression of ethanol-induced behavioral sensitization (^##^*P* ≤ 0.01 and ^###^*P* ≤ 0.001, ethanol *vs.* saline).

Behavioral sensitization was performed as previously described^50–53^. In this test, ethanol is passively administered, *i.e.,* intraperitoneally administered by the experimenter. On the first 3 days of the experiment (habituation, D1-D3; Fig. 3A), the mice were injected with saline (12.5 ml/kg, i.p.) and immediately placed in the center of the actimeter, which was used to record their horizontal locomotor activity for 5 min. The mice were then divided into saline (saline, N = 20) and ethanol (EtOH, N = 27) groups, with similar baseline locomotor activity levels for both groups. The next day (D4), the sensitization procedure started (Fig. 3A). During the 10 days of the induction phase (D4–D13), the mice received daily i.p. injections of either saline or ethanol (2 g/kg). After the last day of sensitization (D13), the mice injected with ethanol were divided into two groups, sham (N = 13) and tDCS (N = 14), and were subjected to the tDCS procedure as previously described (D14: surgery, D21-D25: stimulation; Fig. 3A). Following ten sessions of stimulation, the mice were left undisturbed in their home cages for 7 days. On day 33 (ethanol challenge), all mice received an i.p. injection of 2 g/kg ethanol, and locomotor activity was evaluated (expression phase). Afterward, ethanol challenges were repeated weekly (D40, D47, D54, and D61). To avoid ethanol-induced behavioral sensitization in saline-treated mice across the five ethanol challenges, half of the control animals (N = 10) received ethanol during the first three ethanol challenges, and the other half (N = 10) received ethanol during the last two ethanol challenges.

## Experiment 3: tDCS effects on ethanol-seeking and ethanol-taking behavior

### Animals

Twenty-four female Swiss mice (8 weeks old at the beginning of the experiment; Janvier, France) were trained and tested in operant conditioning chambers.

### Operant conditioning chambers

In this experiment, the consumption of ethanol was voluntary, *i.e.,* the mice controlled their oral consumption. Operant conditioning for ethanol reinforcement was conducted in six standard operant conditioning chambers (30 cm tall × 40 cm long × 36 cm deep, Bioseb, France) housed in sound-attenuated and ventilated cubicles. Each experimental chamber had one clear plexiglass wall on the front side and three opaque panels on the back, left and right sides. The floor consisted of 3-mm-diameter steel bars spaced 1 cm apart. The left and right sides of each chamber were equipped with a nose-poke hole surmounted by a light cue and a liquid distributor placed at the center of the panel. One nose-poke hole was designated as active such that each nose poke made by the animal would trigger the activation of the associated light cue for 2 s and the delivery of 20 μL of ethanol solution (20%). The activation of the light cue for 2 s corresponded to a time-out period in which other nose pokes were not reinforced. The other nose-poke hole was designated inactive, and nose pokes in this hole triggered no event (*i.e*., neither light cue presentation nor ethanol solution delivery), but responses were recorded. The numbers of nose pokes in the active and inactive holes were automatically recorded using PackWin software (Bioseb).

### Habituation to the taste of ethanol

**Figure 4:**
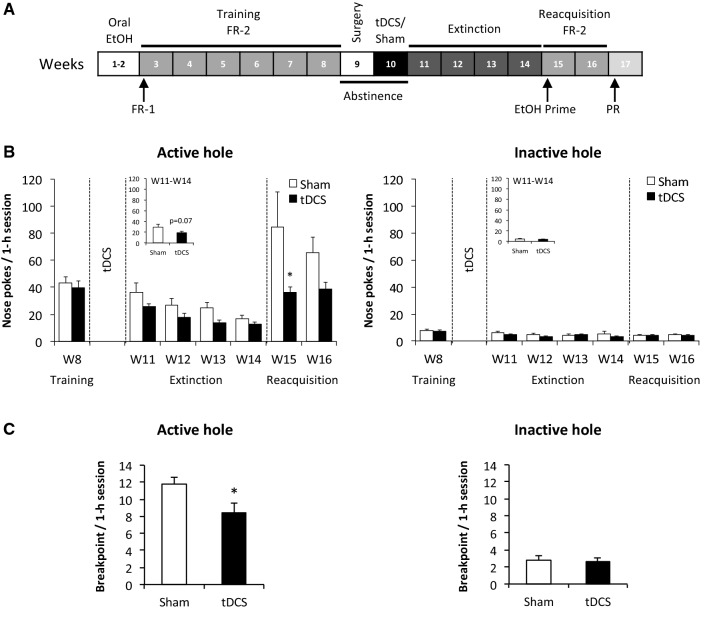
tDCS reduces the reacquisition of ethanol consumption after extinction and the motivation to obtain ethanol in an operant ethanol self-administration paradigm. (**A**) Experimental design. (**B**) Number of nose pokes per session in the active (left) and inactive (right) holes during training before tDCS (week 8, W8) and during extinction (W11-W14) and reacquisition (W15-W16) after tDCS. The average number of nose pokes during extinction (W11-W14) is depicted in the insets (active hole, left; inactive; right). *P ≤ 0.05, tDCS *vs.* sham. (**C**) Motivation to obtain a dose of ethanol, evaluated using a progressive ratio schedule. The breakpoint is lowered in tDCS animals (active hole, left; inactive hole, right). *P ≤ 0.05, tDCS *vs.* sham.

To habituate the mice to the taste of ethanol, they were pre-exposed to increasing concentrations of ethanol in their home cages under a standard two-bottle choice protocol between water and 6%, 12%, and 20% ethanol solution for 3 days, 4 days, and 1 week, respectively (Fig. 4A).

### Operant ethanol self-administration procedure (training)

Each mouse was placed in an operant conditioning chamber and trained to poke the active hole for 20% ethanol solution delivery (20 μL). The mice were initially trained under a fixed ratio 1 (FR-1) schedule of reinforcement for 1 day to allow acquisition of a nose-poke response for ethanol solution. The following day, the response requirement was increased (FR-2) and maintained for 6 weeks (1-h session during the light phase of the day, 5 consecutive days per week (Monday to Friday); Fig. 4A).

After 6 weeks of operant self-administration, the mice were randomly divided into two experimental groups: sham (N = 12) and tDCS (N = 12) groups. Surgery was performed the following week (week 9, W9; Fig. 4A), and repeated tDCS or sham stimulation was applied as described above during week 10 (W10; Fig. 4A). During weeks 9 and 10, the mice were not placed in the operant chambers (abstinence period).

### Operant response during extinction of ethanol self-administration

After tDCS or sham stimulation, the mice were subjected to a period of extinction (W11-W14) during which nose pokes no longer resulted in ethanol solution delivery or light cue presentation. Extinction sessions, similar to training sessions, were conducted during the light phase of the day (1-h session, 5 consecutive days per week; Fig. 4A).

### Reacquisition of operant ethanol self-administration

To trigger memory retrieval of operant responding to ethanol, the light cue and an ethanol prime (20 μL of 20% ethanol) were noncontingently delivered during the first 60 s of the session (W15; first day)^68–70^. Afterward, two nose pokes on the active hole resulted in the delivery of 20 μL of 20% ethanol solution, as described for the training of operant ethanol self-administration (FR-2 schedule). Reacquisition sessions, similar to training sessions, were conducted during the light phase of the day (1-h session, 5 consecutive days per week).

### Progressive ratio schedule

The motivation of the mice to obtain 20% ethanol solution was tested in a single 1-h session (beginning of W17; Fig. 4A) using a progressive ratio schedule in which the sequence of response requirements was increased by a step size of 2 or 3. Animals had to poke the active hole two or three times more than the previous event to receive the next delivery of the ethanol solution. The ratio sequence employed was as follows: 1, 2, 3, 5, 8, 10, 13, 15, 18, 20, 23, 25, 28, 30, 33, 35, 38, 40, 43, and 45. The last ratio completed in the 1-h session was defined as the breakpoint. The higher the breakpoint was, the greater the motivation of the mice to obtain the ethanol solution.

### Statistical analyses

The results are expressed as the mean ± standard error of the mean (SEM). Significance was set at *P* ≤ 0.05. For experiment 1 (CPP), we performed a two-way analysis of variance (ANOVA) with stimulation (sham, tDCS) and dose (0, 1, 2) as between-subject variables. Newman–Keuls (NK) post hoc tests were used to describe differences between individual groups. Student’s t-tests were also used to compare the means of each group with a standard value (*i.e*., a preference score of 0). For experiment 2, regarding the induction of behavioral sensitization, we performed a repeated-measures ANOVA with group (saline, EtOH-sham, EtOH-tDCS) as the between-subject variable and time (D4, D13) as the within-subject variable. For the expression of behavioral sensitization, we used one-way ANOVA with group (saline, EtOH-sham, EtOH-tDCS) as a between-subject variable at each time point (D33, D40, D47, D54, D61). NK post hoc tests were used to describe differences between individual groups. For experiment 3 (ethanol oral self-administration), we performed a repeated-measures ANOVA with stimulation (sham, tDCS) as the between-subject variable and time (W11 to W14, W11 to W16, W8 and W15) as the within-subject variable. NK post hoc tests were used to describe differences between individual groups.

## Results

### Experiment 1: tDCS had no impact on ethanol-induced conditioned place preference

Saline injections did not induce any place preference or aversion in either the sham or tDCS groups (all *P* > 0.05 *vs.* 0, Fig. 2B). In the sham group, ethanol induced a place preference (1 and 2 g/kg, *P* ≤ 0.05 and *P* ≤ 0.01 *vs.* 0, respectively). This response was also observed in tDCS animals (1 and 2 g/kg, *P* ≤ 0.05 and *P* ≤ 0.01 *vs.* 0, respectively). Two-way ANOVA revealed that place preference was modulated by the dose of ethanol (dose effect: F_(2, 48)_ = 20.83, *P* ≤ 0.001). The higher the dose was, the higher the preference score (NK: 0 < 1 g/kg, *P* ≤ 0.001; 1 < 2 g/kg, *P* = 0.08); however, tDCS did not modulate ethanol-induced conditioned place preference (stimulation effect: F_(1, 48)_ = 33.7, *P* = 0.68).

### Experiment 2: tDCS did not modulate the expression of ethanol-induced behavioral sensitization

During the habituation period (D1-D3; Fig. 3A), there were no differences in locomotor activity between the sham and tDCS animals (data not shown). During the first day of the induction of behavioral sensitization (*i.e.,* D4; Fig. 3B), ethanol (2 g/kg) significantly increased locomotor activity compared to saline in both the sham and tDCS groups (treatment x time interaction: F_(1,44)_ = 16.9, *P* ≤ 0.001; D4: EtOH-sham and EtOH-tDCS *vs.* saline, NK: *P* ≤ 0.001 and *P* ≤ 0.01, respectively; Fig. 3B). Ethanol-induced locomotor activity was also observed at the end of the induction phase (*i.e*., D13: EtOH-sham and EtOH-tDCS *vs.* saline, NK: *P* ≤ 0.001). The effect of ethanol on locomotor activity was significantly higher at D13 than at D4 (D13 *vs.* D4: EtOH-sham, *P* ≤ 0.001; EtOH-tDCS, *P* ≤ 0.001), indicating that repeated ethanol injections induced the development of behavioral sensitization in the two experimental groups.

After tDCS treatment (from D21 to D25), the expression of behavioral sensitization was evaluated at D33, D40, D47, D54, and D61 (Fig. 3A). At each time point, a significant group effect was observed (ANOVA for D33, D40, D47, and D54: group effect: *P* ≤ 0.001; ANOVA for D61, group effect: *P* ≤ 0.01). NK post hoc analysis revealed that ethanol induced significantly higher locomotor activity in the animals previously injected with ethanol during the induction phase than in the animals treated with saline (Fig. 3C). This reflected the expression of ethanol-induced behavioral sensitization and showed that this effect was still present at D61, more than 6 weeks after the end of the induction period; however, tDCS had no significant impact on this phenomenon (all *P* > 0.05).

### Experiment 3: tDCS decreased ethanol self-administration

#### Acquisition of operant ethanol self-administration before tDCS

There were no differences during habituation (*i.e*., oral spontaneous consumption, W1 and W2) or during the training period (W3-W8) between the sham and tDCS groups (active hole and inactive hole, all *P* > 0.05; data not shown). During W8 (training pre-tDCS), there was no difference in the number of nose pokes in the active hole between sham and tDCS animals (Student’s t-tests: active hole, *P* > 0.05; inactive hole, *P* > 0.05; Fig. 4B).

#### Extinction of operant ethanol self-administration after tDCS

As expected, the number of nose pokes per session in the active hole decreased from W11 to W14 during the extinction phase (W11-W14, time effect: F_(3,66)_ = 22.3, *P* ≤ 0.001; Fig. 3B). There was a trend toward faster extinction in the tDCS group than in the controls (W11-W14, stimulation effect: F_(1,66)_ = 22.3, *P* = 0.07; Fig. 4B, active hole, inset). No time effect, stimulation effect, or interaction effect was observed for the inactive hole (all *P* > 0.05; Fig. 4B, inactive hole).

#### tDCS attenuated the reacquisition of operant ethanol self-administration

During the first week of reacquisition (W15), the tDCS animals made fewer nose pokes in the active hole than the sham animals (W11-W16, stimulation effect: F_(1,110)_ = 6.80, *P* ≤ 0.05; time effect: F_(5,110)_ = 10.78, *P* ≤ 0.001; stimulation x time effect: F_(5,110)_ = 2.06, *P* = 0.07; NK: W15: sham *vs.* tDCS, *P* ≤ 0.05; Fig. 4B, active hole). This effect was no longer significant the following week (W16, active hole, *P* = 0.13). The number of nose pokes (active hole) was significantly higher in the sham animals during W15 (reacquisition) than during W8 (training pre-tDCS) (W8 *vs.* W15, NK: *P* ≤ 0.05). This was not true in the tDCS animals (*P* > 0.05). No time effect, stimulation effect, or interaction effect was observed for the inactive hole (control condition, all *P* > 0.05; training, extinction, reacquisition).

#### tDCS decreases the motivation to obtain ethanol on a progressive ratio schedule

At the beginning of W17, the breakpoint to obtain a dose of ethanol during the progressive ratio procedure (1-h session) was significantly higher in the sham animals than in the tDCS animals (Student’s t-test: *P*  ≤ 0.05; Fig. 4C, active hole). This effect was not observed for the inactive hole (Student’s t-test: *P* > 0.05; Fig. 4C, inactive hole).

## Discussion

To the best of our knowledge, the beneficial effects of tDCS on alcohol addiction-related behaviors have never been explored in preclinical studies. The goal of the present work was to better identify which phase of alcohol-related behavior (hedonic effect, sensitization, reacquisition after extinction, motivation) might be modulated by repeated anodal tDCS over the frontal cortex in mice. In general, our results indicated that tDCS had no effect when ethanol was passively administered but was very effective in reducing voluntary ethanol consumption. Our data showed that, in contrast to findings with nicotine^43^ and cocaine^44^, tDCS did not modulate the hedonic effect of ethanol assessed in the place preference paradigm, a test that combines Pavlovian conditioning and testing in a drug-free state. Furthermore, tDCS also did not modulate the expression of ethanol-induced behavioral sensitization, which has previously been linked to high ethanol intake in operant procedures^49^. In contrast, tDCS robustly reduced the reacquisition of ethanol consumption (50% decrease) following extinction in an operant paradigm. This is appealing, since this parameter is considered an index of the risk of relapse after a period of abstinence. Furthermore, tDCS significantly decreased the motivation to drink ethanol (30% decrease). That is, the tDCS mice did not work as much as the control (sham) mice to obtain the reward (“wanting” component). These effects were observed more than 1 month after the end of the stimulation period, demonstrating that repeated tDCS has a sustainable impact on these parameters. This is encouraging because the rates of relapse are high in alcohol-dependent patients, even after a long period of abstinence. These results allow us to assume that noninvasive electrical brain stimulation could be useful to help abstinent alcohol-dependent patients avoid relapse.

### tDCS did not modulate ethanol-induced conditioned place preference

Our findings indicated that tDCS had no effect on ethanol-induced conditioned place preference. This is in contrast with our previous data showing that tDCS decreased nicotine-induced conditioned place preference 5 weeks after repeated tDCS (using the same protocol with 0.5 mg/kg nicotine)^43^. Moreover, the increase in nicotine-induced place preference in adults obtained after chronic exposure to nicotine during adolescence was prevented by tDCS^43^. Similarly, cocaine-induced place preference conditioning was reduced 3 weeks after tDCS (for 5 and 25, but not 10, mg/kg)^44^. Regarding ethanol, a dose response was observed in the present study (2 g/kg was more appetitive than 1 g/kg, and 1 g/kg was more appetitive than saline^71,72^), but tDCS did not modulate this parameter. It could be argued, based on Fig. 2B, that the preference score with 2 g/kg ethanol in the tDCS animals decreased and was close to the level of preference observed with 1 g/kg ethanol. However, there was no direct significant difference between the tDCS and sham animals. Moreover, the preference with the 2 g/kg dose of ethanol (compared to a preference score of 0) remained highly robust in the tDCS animals. Clearly, tDCS did not significantly modulate the pleasant effects induced by ethanol (“liking”), in contrast to the modulation observed with other drugs such as nicotine and cocaine. The different mechanisms of action of ethanol compared to psychostimulants might be responsible for these differences. The actions of psychostimulants are limited to a smaller number of neurochemical or receptor systems. Ethanol, on the other hand, interacts with several neurotransmitters in the brain’s reward and stress circuits, which involve multiple receptors at widespread neuroanatomical sites throughout the brain^73^. For example, the rewarding effects of ethanol are mediated both directly and indirectly (e.g., release of GABAergic inhibitory tone and β-endorphin release) on dopaminergic neurons from the ventral tegmental area^74^. Based on the data mentioned above, tDCS seems to have differential outcomes on the hedonic effects of drugs depending on their modes of action on the central nervous system.

### tDCS did not modulate ethanol-induced behavioral sensitization

To date, only one study has explored the impact of tDCS on the locomotor effects of a drug of abuse. In this study, the locomotor activating effects of a high dose of cocaine (25 mg/kg) were reduced by tDCS^44^. The impact of tDCS on behavioral sensitization induced by alcohol and/or other drugs of abuse has never been evaluated. Behavioral sensitization is defined as a progressive and long-lasting increase in specific behaviors after repeated drug exposure, with the most studied behavior being locomotion. A recent study showed that in mice, sensitization to the motor stimulant effects of ethanol was associated with facilitation of the acquisition of ethanol self-administration in an operant task^49^. It was therefore of interest to evaluate the impact of tDCS on ethanol-induced behavioral sensitization. Locomotor sensitization can be divided into two phases: induction and expression. We focused on how tDCS could modulate the expression of behavioral sensitization once the induction was completed. This question has translational value because it would be useful to use tDCS in populations of heavy drinkers who have already been exposed to alcohol. Our data showed that repeated injections of ethanol over a period of 10 days induced, as expected, robust behavioral sensitization in DBA/2 J female mice^50,51,53,75,76^. This phenomenon was still present more than 6 weeks after the end of the induction, demonstrating a long-lasting neuroadaptation to repeated ethanol exposure. However, tDCS had no impact on ethanol-induced behavioral sensitization, at least its expression, in our experimental conditions. Complementary studies are needed to evaluate whether tDCS could preclude the induction of behavioral sensitization induced by ethanol. In this case, tDCS would have to be used as a preventive intervention, which is less practical/relevant for clinical use.

### tDCS reduced the reacquisition of operant ethanol self-administration

The major finding of the present study was the demonstration that repeated anodal tDCS over the frontal lobe can impact addiction-related behaviors in a voluntary oral ethanol self-administration paradigm. Here, tDCS decreased the reacquisition of ethanol self-administration after an extinction period. The number of nose pokes in the active hole was decreased by 50% in the tDCS group compared to the sham group during the first week of reacquisition, 5 weeks after the end of the stimulation period. This suggests that tDCS might decrease the rate of relapse in abstinent alcohol-dependent patients. This is similar to what is observed in humans^19,77,78^. The amount of ethanol intake per session was noticeably higher in sham animals during the first week of reacquisition than in the last week of the training sessions. This was not the case for tDCS animals, which displayed comparable amounts of intake relative to the last week of the training session. Overall, these data indicated that in mice, after an extinction period of 4 weeks, the increase in ethanol consumption that is typically observed was suppressed by tDCS. Therefore, tDCS does not decrease basal ethanol consumption but blocks the increase in ethanol consumption seen after an extinction period (an index of relapse rate). This effect was no longer present the following week due to a less robust effect of extinction on ethanol intake in the sham group at this time point.

We also tested the motivation to consume ethanol with a progressive ratio schedule since elevated motivation is a hallmark of addictive behavior, and we obtained another major significant result regarding ethanol addiction. tDCS decreased the motivation to work to obtain the drug (30% decrease), which indicated a decrease in “drug wanting” (drug craving). Indeed, animals exposed to tDCS displayed a lower breakpoint when tested under a progressive ratio schedule in an operant task. This effect was detected 7 weeks after the end of the stimulation period, which demonstrated a long-term impact of tDCS on this parameter.

These results in mice are in line with an increasing quantity of data obtained from clinical trials. Different laboratories have reported that tDCS reduced craving^19,32–34,40,42^, alcohol consumption^41^, behavioral symptoms associated with alcohol^33,38^ or relapse after gradual withdrawal^19,36,38,40^ in humans. However, others have been unable to replicate these results regarding craving^39^ or relapse^37^. Recent meta-analyses have provided additional evidence that tDCS over the DLPFC reduces craving and ethanol consumption. Interestingly, larger effects were found with repeated stimulations than with single stimulation^77,78^, and a level B (possible efficacy) recommendation has been proposed for tDCS in addiction/craving^79^.

### tDCS decreased “wanting” (motivation) and not “liking” (pleasure)

Taken together, these results suggest a dissociation regarding the effects of tDCS between “liking” (no effect in the CPP) and “wanting” (decrease in ethanol consumption in the self-administration procedure). They also suggest that tDCS is effective when ethanol is voluntarily self-administered and not when it is passively administered, thus revealing that its effectiveness may be more dependent upon motivational aspects. The incentive-sensitization theory posits that the essence of drug addiction is excessive amplification specifically of psychological "wanting" (especially triggered by cues), not necessarily accompanied by an amplification of "liking"^80,81^. Therefore, tDCS might be particularly relevant in the treatment of ethanol-dependent patients and the promotion of abstinence by reducing the wanting component (“craving”). The brain circuitry that mediates the psychological process of "wanting" a particular reward is dissociable from the circuitry that mediates the degree to which it is "liked"^80,81^. Incentive salience or "wanting", a form of motivation, is generated by large and robust neural systems that involve mesolimbic dopamine. By comparison, "liking", the actual pleasurable impact of reward consumption, is mediated by smaller and more fragile neural systems and is not dependent on dopamine. Since tDCS seems to have a pronounced impact on “wanting”, further studies should explore the effect of tDCS on dopamine release induced by alcohol and other drugs of abuse in the nucleus accumbens using in vivo voltammetry and gene induction in corticostriatal circuits^82,83^. We cannot rule out the possibility that both the motivation and hedonic components of alcohol consumption might be affected by tDCS if we used a higher current density and/or more stimulation sessions. However, it is interesting to note that 0.2 mA was sufficient in our previous studies to decrease nicotine- and cocaine-induced place preference, while the exact same protocol of stimulation was not able to decrease ethanol-induced place preference in the present work^43,44^.

Finally, the necessary stimulation parameters clearly differ between humans and rodent models, especially concerning the current density (higher in mice). Additionally, the anatomy of the brain is not the same (different tissue thicknesses between humans and mice, gyrencephalic brain structure in humans vs. lissencephalic structure in mice, etc.). Interestingly, however, the same behavioral parameters are affected by tDCS in clinical and preclinical studies. Indeed, tDCS reduces symptoms associated with depression, improves working memory and attenuates the desire to consume several drugs of abuse both in humans and in mice (see, for example^43,44,46^). Thus, it seems that common mechanisms may be at work in these models and that it is appropriate to study them, bearing in mind that we must be cautious in our conclusions when translating results from animals to humans.

The goal of our study was to establish tDCS in animals to highlight the impact of tDCS on different aspects of ethanol addiction-related behavior. We can now take advantage of this tDCS procedure to better understand the neurobiological mechanisms underlying the behavioral effects of tDCS. Several other questions remain unanswered, notably the duration of tDCS effects on addiction-related behavior. Are they long-lasting effects, or would it be beneficial to add tDCS sessions at different time points to maintain the persistence of its effects? The protocol of the stimulation itself could be improved (e.g., intensity, number of stimulations, interstimulation intervals, position of the electrode, polarity effect, timing of stimulation relative to the extinction procedure), and other stimulation protocols could be tested (e.g., transcranial alternating current and pulsed current stimulation).

## Conclusion

The present study supports the promising clinical results showing that repeated anodal tDCS over the frontal lobe may have beneficial effects on consumption, craving and relapse in users of alcohol (and other drugs of abuse). Our tDCS procedure in animals will allow researchers to better understand the mechanisms of action of tDCS and accelerate its development as an innovative complementary tool to help alcohol-dependent patients maintain abstinence or reduce ethanol intake.
